# Diabetes prevalence and risk factors of early-onset adult diabetes: results from the Indonesian family life survey

**DOI:** 10.1080/16549716.2021.2001144

**Published:** 2021-12-13

**Authors:** Justine Tanoey, Heiko Becher

**Affiliations:** Institute of Medical Biometry and Epidemiology Hamburg, University Medical Center Hamburg-Eppendorf, Hamburg, Germany

**Keywords:** Diabetes, South-East Asia, young adult, childhood, life-course

## Abstract

**Background:**

Diabetes is increasing rapidly in South-East Asia. Studies have reported typical risk factors associated with all-age adult diabetes and highlighted the roles of economic transition and childhood development factors in diabetes in later life. However, little is known about whether these factors were associated with young adult diabetes risk.

**Objectives:**

The study has two main aims: (1) estimate diabetes prevalence among adult participants of the Indonesian Family Life Survey (IFLS), and (2) identify childhood development factors associated with early-onset adult diabetes (diagnosed between age 20 and 40) in Indonesia.

**Methods:**

Data were taken from adults participating in 4^th^ and 5^th^ IFLS (in 2007 and 2014) and linked to childhood history from previous surveys. Diabetes was ascertained from self-report and HbA1c testing in a subsample. Diabetes prevalence rates were estimated by age and source of diagnosis. Cox regression analysis was applied to assess potential risk factors for early-onset adult diabetes.

**Results:**

A total of 34,767 participants were included in the study. Crude total prevalence estimate from self-report was 2,3% and increased with age. Including HbA1c measurements yielded nearly eight times higher prevalence estimates, depending on age. The proportion of yet undiagnosed cases is considerably higher in young age groups. Regression analyses showed that urban childhood residence and high education increased early-onset diabetes risk by 50–70%. Sex, childhood general health, socio-economic level and starvation exposure were not associated with early-onset diabetes risk.

**Conclusion:**

Remarkable differences between diabetes prevalence rates based on self-report and HbA1c measurement indicated the need for better diagnosis, especially in young adults. Urban childhood residence and high education increased early-onset adult diabetes risk. Incorporating these factors and diabetes awareness in existing child health programs, together with screening of individuals at risk, could improve early diabetes detection and prevention strategies among young urban Indonesian adults.

## Background

Indonesia is a lower-middle-income country (World Bank classification) [1] that struggles with the double burden of communicable and non-communicable diseases. The last decades have seen a particular rise in one chronic non-communicable disease, diabetes [2], a condition that is estimated to be among the higher premature mortality causes in South-East Asia, after stroke and coronary heart disease [3], of which the age-adjusted prevalence increased from about 4% in early 1980s to 7% in 2014 [2]. The WHO estimated that diabetes accounted for 6% of all-cause, all-age mortality in Indonesia in 2012 [2,4]. The International Diabetes Federation reported in 2020 that over 6% in approximately 172 million adults in the country suffered from diabetes [5]. According to The Indonesian Basic Health Research in 2013 and 2018, the national prevalence of diabetes (population age 15 and older) has been increasing, and most cases were in age groups of older than 45. A distinction of diabetes types was not clarified as diagnosis was confirmed by self-report, symptoms and blood glucose levels among participants aging 15 and older [6,7].

The reported numbers on diabetes in Indonesia may actually be underestimated as there is a lack of public awareness of its symptoms and risk factors, as well as inadequate diagnosis and treatment available in the public health sector [6,8,9]. In 2013 the Indonesian Ministry of Health reported a crude diabetes prevalence of 6.9% based on blood glucose testing, while according to self-reported diabetes diagnosis, the prevalence was only 2.1%. In addition, approximately a third of the adult population showed pre-diabetes signs of impaired fasting glucose and impaired glucose tolerance [6]. The 2018 report estimated a higher proportion of undiagnosed diabetes and similar rates of individuals with pre-diabetes [7].

Many factors contribute to diabetes manifestation, namely familial history, ethnicity and unhealthy lifestyle [10]. A number of studies have also linked environmental and other exposures to adult diabetes, for example inadequate or excessive gestational weight gain, gestational diabetes, chronic exposure to certain infections and pollutants such as Bisphenol A, iron and nicotine (smoking) [11–16]. Other studies suggested that early-life development factors such as malnutrition during pregnancy or infancy and change from lower-to-higher socio-economic between childhood and adulthood were related to biological modifications resulting in type 2 diabetes in adults [17–21].

Studies in Indonesia have reported several of the known diabetes risk factors, including overweight and obesity, smoking, living in urban areas, and low education [6,22,23]. However, most of these studies did not focus on early-onset adult diabetes (diagnosed between age 20 and 40). One study was found to have observed a small number of young adults showed that unhealthy lifestyle was associated with developing diabetes regardless of family history [24]. In addition, little information could be found beyond the above-mentioned risk factors, particularly early-life and childhood conditions. Revealing the knowledge gap pertaining these young productive individuals may be key to early prevention of the foreseeable tremendous burden caused by diabetes and its comorbidities.

This study has two main aims (i) to investigate the prevalence of diabetes in a large cohort of Indonesians and (ii) to explore childhood development factors potentially associated to the risk of early-onset diabetes.

## Methods

### Data

For this study, we retrieved data from several waves of The Indonesian Family Life Survey (IFLS). The IFLS is a longitudinal cohort study on households and community facilities conducted in 13 out of 27 provinces of Indonesia, where approximately 80% of the population resided at the time of the first survey in 1993. Initially, 22,347 members (including children) of 7,224 households selected by randomized stratified sampling were interviewed. Subsequent surveys in 1997, 2000, 2007 and 2014 followed the same subjects and their expanding families. In the two most recent surveys 44,103 and 50,148 individuals were interviewed, from which data on 29,967 and 36,381 adults, respectively, were included. In these surveys (4^th^ and 5^th^ waves) data collection on diabetes among individuals older than 15 years (considered adults in the survey) began, and in 2014 HbA1c testing on selected individuals (22%) were also added. A detailed description of the IFLS study, the sampling procedure for measurements, and data are available online at https://www.rand.org/well-being/social-and-behavioral-policy/data/FLS/IFLS.html [25–30].

The subjects in this study were adult participants age 15 to 95 who took part in either one or both the diabetes questionnaires in the 4^th^ and 5^th^ surveys. These questionnaires included history of being diagnosed with diabetes, by whom and when, as well as whether prescribed medication (on a weekly basis) was being taken and if insulin injection or other treatment was currently used as therapy. We retrieved socio-demographic information such as birth year, sex and education level, as well as data on childhood developmental factors (based on personal recollections up until age 15) namely, general health condition, history of starvation exposure and parents´ (breadwinner) occupation (based on personal recollections at age 12). Self-reported childhood information was only available from the 5^th^ IFLS. Residence area in childhood was gathered from their mothers, whom were interviewed during the 1^st^ and 2^nd^ IFLS.

The data available from the surveyor website as first linked using individual identity codes to gather all participants and variables of interest from both surveys. The dataset was explored to reveal missing data. Missing values in the study were results of loss to follow-up, interviewers’ error or the participant’s inability to answer. Additionally, missing data in childhood residence area could be because the participants’ mother was not an IFLS participant. Observations with incomplete data were kept, and in variable childhood residence area the missing values were coded as unknown.

Education variables were grouped into low (from no formal education up to elementary school), middle (junior high and high school level) and high (college diploma and above). Information regarding childhood socio-economic level was taken from the reported parent’s occupation (low = unpaid family worker/casual worker, middle = self-employed/permanent employee, high = self-employed with workers). History of starvation data were grouped into yes/no, as age-specific grouping resulted in few occurrences under age 5. Height and weight measurements were used to calculate BMI, and the earlier value was chosen if available. The BMI variable was categorized according to the WHO Western Pacific recommendation, and missing values were coded as unknown [31].

### Outcome ascertainment

The outcome of interest, diabetes, was obtained by self-report and when available, by HbA1c (glycated haemoglobin) blood test result. During the 5^th^ IFLS an HbA1c test was performed on a sample of study participants, regardless of their claimed diabetes status [27,28]. The sampling method initially began in 2007 (IFLS 4) with taking blood samples from interviewed individuals aged 1 or older in a random half of the original households (IFLS 1) and its split-offs (former family members having their own households), intended to be tested for C-Reactive Protein (a biomarker for systemic inflammation). Then a sampling algorithm based on different age groups and same household was applied, with priority selection of those aged 50 and older. At the IFLS 5, blood samples from the same individuals were taken and measured for HbA1c. The method applied in the testing used dried blood spot samples, for which the surveyors had repeated validation checks showing consistent results to whole blood testing (correlation R^2^ range = 0.985–0.994). Individual sampling selection and the conversion algorithm to equivalent whole blood testing values are also available in the survey’s working paper [27,28]. An individual was considered to have diabetes when they reported ever being diagnosed with diabetes at both or at the 5^th^ survey, or when their HbA1c value was ≥6.5%, if tested [32].

### Statistical analysis

Age-specific diabetes prevalence in the cohort was estimated for the year 2014 based on age at that survey. For comparison, we calculated prevalence estimates based solely on self-report and estimates which included individuals with previously unknown diabetes and HbA1c measurement ≥ 6.5%.

Age at diagnosis was based on self-report or age at the 5^th^ IFLS if diagnosis was established by HbA1c test during the survey. Female participants who were pregnant and diagnosed with diabetes at the same year of the survey were regarded as suspected gestational diabetes. These cases, as well as subjects younger than age 20 were included in the prevalence estimates.

To investigate the potential risk factors associated with early-onset diabetes, we applied Cox proportional hazard regression in uni- and multivariable analyses. An event was defined as self-reported diabetes diagnosis or blood-tested HbA1c level ≥ 6.5% between age 20 and 40 years. Observation time was time since 20^th^ birthday. Individuals below age 20 were thus excluded in this analysis. Individuals who reported having diabetes before age 20 were also excluded as they were likely to have been type 1 diabetes. End of observation time was defined as age at diagnosis for events or 40^th^ birthday for non-events, whichever came first. Risk factors of interest were sex, education and childhood conditions i.e. socio-economic level, residential area, general health and starvation exposure. We did not employ a formal variable selection procedure such as backward elimination but rather selected the variables by prior knowledge. We stratified the multivariable model analyses by birth year to reduce bias due to overrepresentation of diabetes prevalence in those born in later decades, and included the household identity code as a cluster variable to adjust for familial diabetes.

In plausibility checks and sensitivity analyses we (i) performed logistic regression analysis to investigate whether being tested for HbA1c was associated with age, sex, BMI and self-reported diabetes, (ii) excluded individuals aged 20 to 40 who had no HbA1c measurement since among this age group the diabetes cases among those who had no HbA1c measurement remained undetected, (iii) excluded individuals who developed diabetes after age 40 as it was possible that they were early-onset diabetes that were late-diagnosed and (iv) applied only self-reported diabetes to simulate an actual situation without screening. In addition, we (v) excluded individuals who had BMI of ≤ 23 kg/m^2^ and (vi) who also reported taking insulin as treatment. Furthermore, we (vii) included BMI in the Cox regression multivariable model.

All analyses were performed in R [33].

## Results

**Figure 1.**
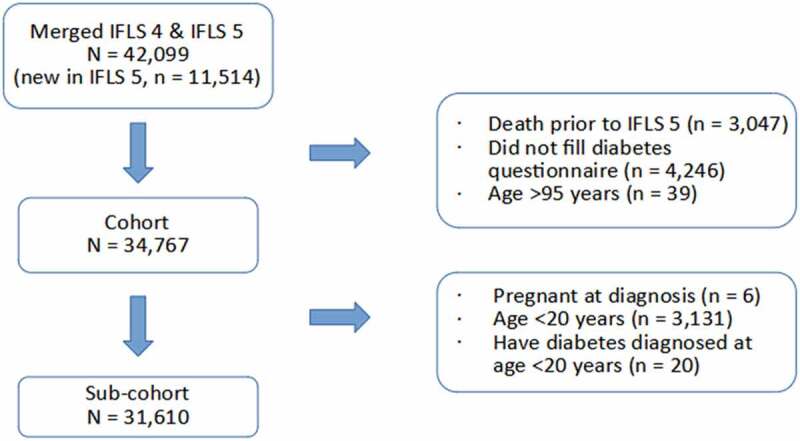
Indonesian family life survey: Number of participants in present study following inclusion and exclusion criteria

A total of 34,767 participants remained after exclusion due to non-participation in both diabetes questionnaires and death prior to the 5^th^ IFLS (Figure 1.). Description of individuals with or without diabetes among the study participants in relation to age distribution at the 5^th^ IFLS are described in Table 1. Diagnoses were confirmed by self-report and in part also by HbA1c test. There were 6.699 participants tested for HbA1c included in the analysis, and 8.4% were found to have levels ≥ 6.5% (age group details shown in Supplement Table S1). Approximately half of the participants who admitted having diabetes and had their HbA1c level tested stated they were not taking prescription medicine. High levels (≥ 7%) of HbA1c were found in a proportion of those who stated they were taking prescription medicine (details shown in Supplement Table S2). Individuals with a positive diagnosis admission or a positive HbA1c test for diabetes were considered diabetes cases. The prevalence increased with age, where more than 50% of diabetes cases were found in participants older than 50 years.

**Table 1.** Indonesian family life survey: Distribution of diabetes cases (based on self-report and HbA1c test) and prevalence estimates in the study cohort by age

Self-reported Diabetes^§^	HbA1c Test^#^	Age groups*						Total
		15–19	20–30	31–40	41–50	51–60	> 60	
+	≥ 6.5%	0	1	2	13	46	58	120
+	< 6.5%	1	1	7	4	14	24	51
+	NA	1	21	83	140	201	179	625
–	≥ 6.5%	10	29	69	73	97	162	440
–	< 6.5%	515	1340	1297	776	784	1376	6088
–	NA	2616	6919	7462	5009	3101	2336	27,443
**Total tested**		526	1371	1375	866	941	1620	6699
**Total non-tested**		2617	6940	7545	5149	3302	2515	28,068
**Total**		3143	8311	8920	6015	4243	4135	34,767
**Prevalence estimates, based on**	**self-report** **(among all)**	0.1%	0.3%	1.0%	2.6%	6.2%	6.3%	2.3%
	**self-report (among tested)**	0.2%	0.1%	0.7%	2.0%	6.4%	5.1%	2.6%
	**self-report or HbA1c ≥ 6.5%** **(among tested)**	2.1%	2.3%	5.7%	10.4%	16.7%	15.1%	9.1%

* Age (in years) in 2014. ^**§**^*Self-reported diabetes summarized from overall ‘Diabetes Diagnosis’ variable.* *^#^ At the 5^th^ IFLS HbA1c test was first performed on a sample of a participants (level ≥6.5% = diabetes positive).*

Table 1 demonstrates that a large proportion of diabetes cases were unknown to the individual, as seen in the proportion of all self-reported and HbA1c detected diabetes among tested individuals in comparison to those who only self-reported diabetes. The discrepancies were more pronounced in younger ages. For example, in the age group 31–40 years the prevalence of self-reported diabetes among all and among tested individuals were 1.0% and 0.7%, respectively. However, If we added the individuals with increased HbA1c levels as diabetes cases, the prevalence estimate was 5.7%, thus being about six times higher. Comparatively, in the age group 51–60 years the numbers were 6.2%, 6.4% and 16.7%, making the ratio only 2.6 times higher.

**Figure 2.**
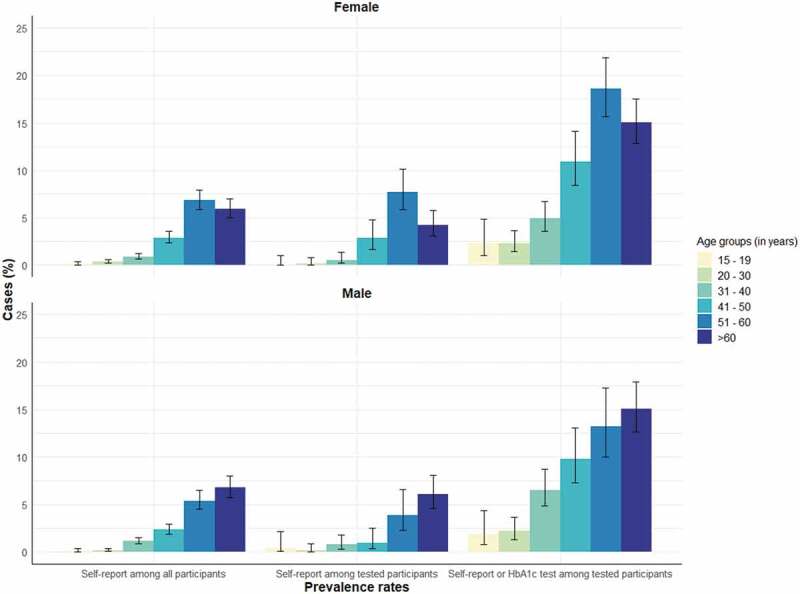
Indonesian family life survey: Diabetes prevalence estimates in the cohort according to self-report and HbA1c measurement in different age groups and each sex

Figure 2 illustrates the prevalence estimates according to source of diagnosis among all participants and those who underwent HbA1c screening by sex. The proportions of self-reported cases between both females and males were similar. Both Table 1 and Figure 2 show that the cases found through HbA1c testing added considerably to the total prevalence in the cohort across all age groups for both males and females.

Several characteristics of the sub-cohort by case status are described in Table 2. These characteristics were analysed in uni- and multivariable Cox proportional hazard regression models and the risk estimates are shown in Table 3. In univariable analysis, significant associations between education, childhood socio-economic level and residence area and early-onset diabetes risk were observed. No sex difference was seen, and neither was any association between general childhood condition nor exposure to starvation in childhood. Multivariable Cox model analysis with sex, education and childhood condition factors included 28,312 observations with 257 cases. It showed that accounting for all the variables, high education and urban residence area in childhood remained significant risk factors in increasing the risk of early-onset diabetes by 50–70%.

**Table 2.** Indonesian family life survey: Cohort characteristics defined by diabetes status and age at diagnosis (in years)

Variable		Diabetes (age 20–40) (N = 273)	Diabetes (age > 40) (N = 937^^^)	No diabetes (N = 30,400)	Total^§^ (N = 31,610)
**Birth year**	Before 1960	14 (5.1%)	609 (65.0%)	5374 (17.7%)	5997 (19.0%)
	1960–1969	22 (8.1%)	259 (27.6%)	4904 (16.1%)	5185 (16.4%)
	1970–1979	122 (44.7%)	69 (7.4%)	7072 (23.3%)	7263 (23.0%)
	1980 − 1989	97 (35.5%)	0 (0.0%)	8900 (29.3%)	8997 (28.5%)
	1990 onwards	18 (6.6%)	0 (0.0%)	4150 (13.7%)	4168 (13.2%)
**Sex**	Female	139 (50.9%)	527 (56.2%)	15,868 (52.2%)	16,534 (52.3%)
	Male	134 (49.1%)	410 (43.8%)	14,532 (47.8%)	15,076 (47.7%)
**Education level**	Low	71 (26.0%)	501 (53.5%)	11,608 (38.2%)	12,180 (38.6%)
	Middle	148 (54.2%)	308 (32.9%)	14,467 (47.7%)	14,923 (47.3%)
	High	54 (19.8%)	127 (13.6%)	4281 (14.1%)	4462 (14.1%)
**General childhood health condition**	Poor	22 (8.6%)	65 (8.1%)	2100 (7.7%)	2187 (7.7%)
	Fair	64 (24.9%)	200 (24.8%)	8030 (29.4%)	8294 (29.3%)
	Good	108 (42.0%)	322 (40.0%)	10,889 (39.9%)	11,319 (39.9%)
	Very good	43 (16.7%)	153 (19.0%)	4886 (17.9%)	5082 (17.9%)
	Excellent	20 (7.8%)	66 (8.2%)	1379 (5.1%)	1465 (5.2%)
**Ever experienced starvation in childhood**	No	237 (92.2%)	685 (85.0%)	25,002 (91.6%)	25,924 (91.5%)
	Yes	20 (7.8%)	121 (15.0%)	2282 (8.4%)	2423 (8.5%)
**Childhood** **socio-economic level**	Low	32 (12.5%)	98 (12.2%)	3911 (14.3%)	4041 (14.3%)
	Middle	145 (56.4%)	408 (50.6%)	13,183 (48.3%)	13,736 (48.5%)
	High	80 (31.1%)	300 (37.2%)	10,191 (37.4%)	10,571 (37.3%)
**Residential area in childhood**	Urban	11,335 (56.6%)	75 (42.1%)	324 (42.2%)	11,734 (56.0%)
	Rural	8677 (43.4%)	103 (57.9%)	443 (57.8%)	9223 (44.0%)

^^^Includes diabetes patients with unknown age at diagnosis (n = 15). *^§^ Individuals without diabetes age <20, individuals who reported having diabetes diagnosed at age <20 and women who developed diabetes during pregnancy were excluded.*

**Table 3.** Indonesian family life survey: *Uni- and multivariable Cox regression analyses on sex and childhood conditions, Indonesia <1993 – 2014 > .*

Outcome: Early-onset Diabetes	Levels	n (%)	HR *^§^* (Univariable)	HR *^§^* (Multivariable)^°^
**Sex**	Female	16,534 (52.3)	1	1
	Male	15,076 (47.7)	1.03 (0.81–1.30, p = 0.83)	0.98 (0.77–1.25, p = 0.883)
**Education level**	Low	12,180 (38.6)	1	1
	Middle	14,923 (47.3)	2.67 (2.01–3.55, **p < 0.001**)	1.34 (0.95–1.87, p = 0.094)
	High	4462 (14.1)	3.49 (2.45–4.98, **p < 0.001**)	1.67 (1.10–2.52, **p = 0.015**)
**General health in childhood**	Poor	2187 (7.7)	1.00 (0.63–1.58, p = 0.999)	1.11 (0.69–1.80, p = 0.666)
	Fair	8294 (29.3)	0.83 (0.61–1.13, p = 0.237)	0.82 (0.60–1.11, p = 0.199)
	Good	11,319 (39.9)	1	1
	Very good	5082 (17.9)	0.95 (0.66–1.35, p = 0.762)	0.85 (0.60–1.21, p = 0.375)
	Excellent	1465 (5.2)	1.34 (0.83–2.16, p = 0.232)	1.48 (0.92–2.39, p = 0.105)
**Ever experienced starvation in childhood**	No	25,924 (91.5)	1	1
	Yes	2423 (8.5)	0.67 (0.42–1.05, p = 0.083)	1.23 (0.76–1.99, p = 0.397)
**Childhood socio-economic level**	Low	4041 (14.3)	0.72 (0.49–1.05, p = 0.088)	0.87 (0.58–1.29, p = 0.488)
	Middle	13,736 (48.5)	1	1
	High	10,571 (37.3)	0.65 (0.49–0.85, **p = 0.002**)	0.82 (0.62–1.09, p = 0.165)
**Residence area in childhood**	Rural	11,734 (37.1)	1	1
	Urban	9223 (29.2)	1.67 (1.24–2.25, **p = 0.001**)	1.51 (1.08–2.10, **p = 0.016**)
	Unknown	10,653 (33.7)	1.59 (1.17–2.15, **p = 0.003**)	0.97 (0.71–1.33, p = 0.853)

*^§^ HR – hazard ratio stratified by birth year and include household as clusters.* *^°^ Multivariable model: n = 28,312; n cases = 257.*

Logistic regression analysis to assess whether certain characteristics influenced the likelihood of being tested for HbA1c showed positive association to older age (OR 1.03, 95% CI [1.02–1.03]), negative associations with being male (OR 0.87, 95% CI [0.82–0.92]) and having higher BMI (OR 0.98, 95% CI [0.97–0.98]). However, there was no association with self-reported diabetes (OR 0.84, 95% CI [0.70–1.01]). Sensitivity analyses results (Supplement Table S3) from similar multivariable model with cases diagnosed after age 40 excluded or with only self-reported cases applied in the sub-cohort showed similar risk associations in a slightly larger magnitude. Analyses excluding participants who either had BMI ≤ 23 kg/m^2^ or also taking insulin as treatment resulted in case reductions (78 and 5, respectively), but yielded similar results (Supplement Table S4). The addition of BMI in the multivariable Cox regression model showed that BMI was significantly associated with early-onset adult diabetes risk and the effects estimates of other covariates were in a similar order of magnitude (Supplement Table S5).

## Discussion

This study utilized the longitudinal IFLS survey data that provided health information on a relatively large cohort with samples that represented 83% of the Indonesian population in 1993 [25,34]. The 2014 IFLS applied HbA1c blood testing to measure blood glucose levels, whose value reflects its history for the past 3 months and may be used for diabetes diagnosis as well as monitoring [35–38]. Our estimates showed that self-reported diabetes prevalence in the cohort aged between 15 to 95 years old was 2.3%. HbA1c testing performed on a sample of participants in the 2014 IFLS revealed 8.4% diabetes prevalence, including 440 participants with undiagnosed diabetes (6.6% of tested individuals), which contributed a substantial third of the total prevalence in the cohort. This prevalence rate of undiagnosed diabetes found through screening was relatively similar to that reported in the 2013 Indonesian Basic Health Research [6]. Prevalence estimate based on self-reported diabetes was higher than the 2013 national report [6], possibly due to higher presentation of urban residents in the IFLS, who have more access to better healthcare. However, this and the prevalence estimate among HbA1c tested participants were similar to the 2018 Indonesian Basic Health Research estimates [7], confirming an increasing trend over the years [7]. Furthermore, profoundly higher diabetes prevalence rates found through random testing compared to self-report demonstrated in this study, particularly in the younger age group (under 50), emphasized the lack of awareness of the disease, as has been reported in other studies [39,40], and highlighted the potential benefit of screening individuals at-risk.

Most of the diabetes cases in the cohort were diagnosed at nearly 50 years of age, similar to reports about the typically younger age of onset of diabetes among Asians compared to Caucasians [12,41]. However, about one third of the cases were found by random sample HbA1c testing, so the cohort mean age at diagnosis could be older if testing had been done after symptoms or complications emerged. Nevertheless, the presence of heightened HbA1c levels indicated an ongoing diabetic condition. This is a particularly alarming signal because earlier onset diabetes carries a higher impact on comorbidities and life quality of the individuals resulting from the longer disease duration and possibly more aggressive course [42–46]. In addition, only half of the participants with diabetes reported consuming prescribed medication and HbA1c screening showed persisting high levels in a number of tested participants with diabetes. These pointed out the necessity for regular monitoring to ensure that patients received adequate therapy and complied to their treatment regiment, in order to prevent premature mortality and other complications [36,47].

Interestingly, the findings that approximately third of diabetes patients were previously undiagnosed, and approximately half were being treated somewhat fit the ‘rule of halves’ for chronic diseases in the community proposed in the early 1970’s. In case of this phenomenon, measures to improve case finding, management and monitoring would require organized efforts and substantial cost, but indispensable to achieve significant disease reduction [48].

Subsequent analyses focusing on early-onset diabetes risk indicated that high education and urban residential area in childhood posed higher risks for the young adults. The finding that high education was related to increased diabetes risk is in line with the concept of stage 1 obesity epidemiology transition [49] and results from other studies showing that in low- and middle-income countries, in contrast to high-income countries [50], higher education was pertinent to higher type 2 diabetes risk [51]. Better education in developing countries could mean higher socio-economic status and improved life quality, but this could also present health disadvantages due to the change to more sedentary jobs and animal-based nutrition. Furthermore, living in urban areas exposed residents to faster economic development changes that could increase diabetes risk by promoting overweight and obesity, such as more access to unhealthy fast-foods, sedentary lifestyle (less walking and more usage of motorized vehicles), imbalanced diet (high energy-dense foods) and environmental pollution [12,18,41,52]. Our analysis showed that these exposures were not only important as adults, but also as children. It is likely that such early and prolonged exposure assisted in establishing unhealthy lifestyle and accumulated risk as adults.

Self-reported childhood general health, exposure to starvation and socio-economic level were not associated with early-onset diabetes risk in this cohort. Exposure to starvation in childhood showed a protective effect tendency in univariable analysis, but the opposite when fit in a multivariable model. Since the variance of these estimates was large, further interpretation was not possible. Experiencing malnutrition in utero and in infancy and then being able to obtain better nutrition in later life have been associated with higher diabetes risk in later life [53–55]. Low childhood socio-economic status also might increase diabetes risk as adults [56], although other evidence has shown that adult socio-economic status could be more relevant [57,58]. In this cohort the information was based on the subjects’ recollection as a child, so the result could be affected by recall bias. In addition, the status was estimated based solely on type of parent occupation that did not allow for further distinction in household income as another socio-economic indicator. The age of starvation exposure was also not considered in the analysis, and this could well have obscured any existing effect of early-life starvation. Furthermore, the change to higher socio-economic status in later life, which could better capture the influence on diabetes manifestation, could not be ascertained from the data.

In the multivariable model, high compared to low education and urban childhood residence area remained significant risk factors for early-onset diabetes. Each of this aspect contributed approximately 60% larger risk in the young adults. These results supported the notion that childhood conditions had important roles in diabetes development in Indonesia. It underlined the risks for young urban dwellers and highlighted the opportunity for targeted diabetes intervention and prevention measures, as previous studies have indicated geographical variability in diabetes prevalence in Indonesia [59]. In 2015, the government set a national policy to tackle the increasing number of non-communicable diseases including diabetes by establishing primary and secondary prevention (disease management) programs at local government health clinics and health promotion campaigns [60]. However, operations have been hindered by inadequate staff training and constrained resources [61,62]. Taking the findings in this study into account could assist in prioritizing regions where the population was most vulnerable for strategic resources allocation. Moreover, recognizing early-life developmental factors as risk factors would facilitate risk reduction at an earlier stage, particularly for factors that could lead to unhealthy behaviour and more challenging to modify once ingrained [63]. Therefore, it would also be prudent and effective to incorporate diabetes awareness in existing mother and child health programs in the country.

Our sensitivity analyses demonstrated some differences in risk estimates depending on whether the cohort was restricted to only HbA1c level-tested individuals. It is likely that the discrepancy is caused by the much smaller number of tested individuals and cases. However, it is also possible that the higher risk estimates seen in the whole cohort was driven by larger number of self-reported cases by participants living in urban areas. Further studies with more comprehensive laboratory confirmed diabetes diagnosis, including C-peptide level to distinguish type 1 diabetes [64], is warranted to investigate these associations.

There are limitations to this study that need to be addressed. Firstly, the secondary data analyzed here were not specifically designed to assess the risk of diabetes in relation to early-life or childhood developmental factors. This presented certain challenges, one being missing values in several variables of interest, most notably those collected from the mothers of the subjects. The missing values were unlikely to be related to the outcome. However, substantial bias may exist and it needs to be taken in consideration when interpreting the findings. In addition, the presence of high BMI prior to diagnosis, a major diabetes risk factor, could not be clarified in all participants. Our sensitivity analysis with available BMI values in the multivariable model indicated that BMI could likely be a modifier in the pathway linking high education and childhood urban residence with early-onset diabetes. Another challenge was that the majority of cases were based on self-report and it was not possible to ascertain the diabetes type. We assumed that most cases in our risk analysis were type 2 diabetes, as we excluded those diagnosed before age 20 and there were extremely low prevalence rates of other diabetes types ever reported in Indonesia and the region [65–67]. Moreover, the true number of cases in the cohort is likely to be higher than reported here, and this might have caused bias in the risk estimates. Lastly, the blood HbA1c testing applied for diagnostic criteria could be influenced by factors such as certain drugs and illnesses [68]. However, the extent of any effects was beyond the scope of this study.

## Conclusion

Based on a large adult cohort, this study demonstrated evidence that there was a consequential issue of diabetes in Indonesia, where most cases remained presumably undiagnosed. In a country with more than 170 million adults, an approximated prevalence of 8% would tremendously add to the local and global diabetes burden of disease. Increasing awareness of the illness and its risk factors in both health providers and the public, as well as targeted screening on individuals at-risk could improve timely diagnosis and prevent severe complications. To the best of our knowledge, this is the first study that applied HbA1c measurement to estimate diabetes, including undiagnosed, prevalence and subsequently investigated childhood development factors as potential early-onset adult diabetes risk factors in a large Indonesian cohort. The findings here showed that living in urban areas as children, together with high education, increased the risk of early-onset diabetes. In a country where resources are limited and diabetes prevalence vary geographically, integrating these findings into existing diabetes control and child health programs could assist in directing their efforts into areas where they would be most effective and efficient. In addition, the IFLS has integrated childhood data collection into their survey, and although detailed early-life and specific-disease pertinent information was somewhat limited, it would be a major advantage for researchers to utilize such a readily available large dataset. The results could ideally support life-course public health actions towards mitigating the consequent burden of non-communicable diseases.

## Supplementary Material

Supplemental Material

## Data Availability

The data that support the findings of this study are available at https://www.rand.org/well-being/social-and-behavioral-policy/data/FLS/IFLS.html.

## Paper context

Diabetes is a growing major public health issue in Indonesia. Our study found a large discrepancy between prevalence rates by HbA1c screening (8.4%) and self-report (2.5%), and the differences are greater the younger the age. Furthermore, we identified living in urban areas as children and high education as risk factors for early-onset adult diabetes. Incorporating these findings into existing diabetes control and child health programs could improve the success of early prevention in risk groups.

## References

[1] New world bank country classifications by income level: 2021-2022 [Internet]. World Bank Group. 2021 cited 2021 Sep 07. Available from: https://blogs.worldbank.org/opendata/new-world-bank-country-classifications-income-level-2021-2022.

[2] World Health Organization. Indonesia Diabetes Country Profile. 2016. cited 2019 Apr 23. Available from: https://www.who.int/diabetes/country-profiles/idn_en.pdf.

[3] Cardiovascular Division & Health Services Research Centre. Reducing the burden of CVD in Indonesia. Newtown: The George Institute for Global Health; 2017. cited 2019 Feb 1]. Available from https://www.georgeinstitute.org/sites/default/files/reducing-the-burden-of-cvd-in-indonesia-evidence-review.pdf

[4] World Health Organization. WHO diabetes country profiles 2016: explanatory notes. 2016. cited 2019 Feb 18. Available from: https://www.who.int/diabetes/country-profiles/diabetes_profiles_explanatory_notes.pdf?ua=1.

[5] International Diabetes Federation. Indonesia: IDF; 2020 [updated 2020 May 14; cited 2021 Feb 28]. Available from: https://www.idf.org/our-network/regions-members/western-pacific/members/104-indonesia.

[6] Indonesia Agency of Health Research and Development. Basic health research (Riskesdas) 2013. Indonesia: Ministry of Health Republic of Indonesia; 2013. cited 2019 Mar 25]. Available from https://www.kemkes.go.id/resources/download/general/Hasil%20Riskesdas%202013.pdf

[7] Indonesia Agency of Health Research and Development. Basic health research (Riskesdas) 2018. Jakarta Indonesia: Ministry of Health of Republic of Indonesia; 2019. cited 2020 Nov 6. Available from: https://www.litbang.kemkes.go.id/laporan-riset-kesehatan-dasar-riskesdas/.

[8] Arisma BJN, Yunus M, Fanani E. [Description of community knowledge on diabetes mellitus risk factors in sub-district Pakisaji in Malang district]. Preventia. 2017;2:2.

[9] Gultom YT [Diabetes mellitus patients’ knowledge on diabetes management at the gatot soebroto army hospital in central jakarta] [thesis]. Depok Indonesia: University of Indonesia; 2012.

[10] Chan JC, Yeung R, Luk A. The Asian diabetes phenotypes: challenges and opportunities. Diabetes Res Clin Pract. 2014;105:135–11. Epub 2014 Jun 21. PubMed PMID: 24947419. 24947419 10.1016/j.diabres.2014.05.011

[11] Barker DJ. Fetal growth and adult disease. Br J Obstet Gynaecol. 1992;99:275–276. Epub 1992 Apr 1. PubMed PMID: 1581269 1581269 10.1111/j.1471-0528.1992.tb13719.x

[12] Chan JC, Malik V, Jia W, et al. Diabetes in Asia: epidemiology, risk factors, and pathophysiology. JAMA. 2009;301:2129–2140. PubMed PMID: 19470990 Epub 2009 May 28. 19470990 10.1001/jama.2009.726

[13] Jiang X, Ma H, Wang Y, et al. Early life factors and type 2 diabetes mellitus. J Diabetes Res. 2013;2013:485082. Epub 2014 Jan 24. PubMed PMID: 24455747; PubMed Central PMCID: PMCPMC3876901. 24455747 10.1155/2013/485082 PMC3876901

[14] Ma RC, Chan JC, Tam WH, et al. Gestational diabetes, maternal obesity, and the NCD burden. Clin Obstet Gynecol. 2013;56:633–641. Epub 2013 Jul 4. PubMed PMID: 23820121. 23820121 10.1097/GRF.0b013e31829e5bb0

[15] Sarr O, Yang K, Regnault TR. In utero programming of later adiposity: the role of fetal growth restriction. J Pregnancy. 2012;2012:134758. Epub 2012 Dec 20. PubMed PMID: 23251802; PubMed Central PMCID: PMCPMC3518064. 23251802 10.1155/2012/134758 PMC3518064

[16] Tam CHT, Ma RCW, Yuen LY, et al. The impact of maternal gestational weight gain on cardiometabolic risk factors in children. Diabetologia. 2018;61:2539–2548. 30225524 10.1007/s00125-018-4724-x PMC6223878

[17] Kadayifci FZ, Haggard S, Jeon S, et al. Early-life programming of type 2 diabetes mellitus: understanding the association between epigenetics/genetics and environmental factors. Curr Genomics. 2019;20:453–463. PubMed PMID: 32477001 32477001 10.2174/1389202920666191009110724 PMC7235385

[18] Nanditha A, Ma RC, Ramachandran A, et al. Diabetes in Asia and the Pacific: implications for the global epidemic. Diabetes Care. 2016;39:472–485. PubMed PMID: 26908931 Epub 2016 Feb 26. 26908931 10.2337/dc15-1536

[19] Uma SV, Raman VK, Nochikattil SK. Life course socioeconomic transition and its association with early onset type 2 diabetes: protocol for a sequential exploratory mixed method study. J Clin Diagn Res. 2016;10:LO01–LO5. PubMed PMID: 27504317 27504317 10.7860/JCDR/2016/17800.8020 PMC4963677

[20] Vaag A, Brøns C, Gillberg L, et al. Genetic, nongenetic and epigenetic risk determinants in developmental programming of type 2 diabetes. Acta Obstet Gynecol Scand. 2014;93:1099–1108. PubMed PMID: 25179736 Epub 2014 Sep 3. 25179736 10.1111/aogs.12494

[21] Vaiserman AM. Early-life nutritional programming of type 2 diabetes: experimental and quasi-experimental evidence. Nutrients. 2017;9:236. PubMed PMID: 28273874 10.3390/nu9030236 PMC5372899 28273874

[22] Indrahadi D, Wardana A, Pierewan A. The prevalence of diabetes mellitus and relationship with socioeconomic status in the Indonesian population. J Gizi Klinik Indones. 2021;17:103.

[23] Soewondo P, Pramono LA. Prevalence, characteristics, and predictors of pre-diabetes in Indonesia. Med J Indones. 2011;20:4. 21063043

[24] Antono L, Kurniati A, Angela A, et al. The incidence of diabetes in Indonesian young adults: the importance of lifestyle compared to family history. International Conference on Food and Agricultural Sciences (ICFAS 2013); 5-6 Oct 2013; Melaka, Malaysia: IPCBEE; 2013.

[25] Frankenberg E, Karoly L. The 1993 Indonesian family life survey: overview and field report. RAND Labor & Population, 1995 Nov 1995. Report No.: DRU-1195/1-NICHD/AID Contract No.: DRU-1195/1-NICHD/AID.

[26] Frankenberg E, Thomas D The Indonesia family life survey (IFLS): study design and results from waves 1 and 2. RAND Labor & Population, 2000 Mar 2000. Report No.: DRU-2238/1-NIA/NICHD Contract No.: DRU-2238/1-NIA/NICHD.

[27] Herningtyas EH, Hu P, Edenfield M, et al. IFLS wave 5 dried blood spot data user guide. Santa Monica: RAND Labor & Population, 2018 Jan. WR-1143/6-NIA/NICHD Contract No.: WR-1143/6-NIA/NICHD.

[28] Hu P, Herningtyas EH, Strauss J, et al. IFLS C-Reactive protein data user guide. Santa Monica: RAND Labor & Population, 2013 Oct. Contract No.: WR-675/7.

[29] Strauss J, Witoelar F, Sikoki B The fifth wave of the Indonesia family life survey (IFLS5): overview and field report. RAND Labor & Population, 2016 Mar. WR-1143/1-NIA/NICHD Contract No.: WR-1143/1-NIA/NICHD.

[30] Strauss J, Witoelar F, Sikoki B, et al. The fourth wave of the Indonesia family life survey (IFLS4): overview and field report. RAND Labor & Population, 2009 Apr. WR-675/1-NIA/NICHD Contract No.: WR-675/1-NIA/NICHD.

[31] World Health Organization. Regional office for the Western Pacific. In: The Asia-Pacific perspective: redefining obesity and its treatment. Sydney: Health Communications Australia; 2000. p. 55.

[32] American Diabetes Association. Introduction: standards of medical care in diabetes-2020. Diabetes Care. 2020;43:S1–s2. Epub 2019/ 12/22. PubMed PMID: 31862741. 31862741 10.2337/dc20-Sint

[33] R Core Team. R: a language and environment for statistical computing. 4.0.3 ed. Vienna Austria: R Foundation for Statistical Computing; 2020.

[34] RAND. The Indonesia family life survey (IFLS): RAND; 2015 [cited 2018]. Available from: https://www.rand.org/well-being/social-and-behavioral-policy/data/FLS/IFLS.html.

[35] American Diabetes Association. 2. Classification and diagnosis of diabetes: standards of medical care in diabetes—2021. Diabetes Care. 2021;44:S15–S33. 33298413 10.2337/dc21-S002

[36] American Diabetes Association. 6. Glycemic targets: standards of medical care in diabetes—2020. Diabetes Care. 2020;43:S66–S76. 31862749 10.2337/dc20-S006

[37] American Diabetes Association. 12. Older adults: standards of medical care in diabetes—2020. Diabetes Care. 2020;43:S152–S62. 31862755 10.2337/dc20-S012

[38] Sherwani SI, Khan HA, Ekhzaimy A, et al. Significance of HbA1c test in diagnosis and prognosis of diabetic patients. Biomark Insights. 2016;11:95–104. PubMed PMID: 27398023 27398023 10.4137/BMI.S38440 PMC4933534

[39] Kristina S, Salsabila A, Hanifah S. Awareness of diabetes mellitus among rural population in Indonesia. Int J Pharm Res. 2020; 13. 10.31838/ijpr/2021.13.01.027.

[40] Ligita T, Wicking K, Francis K, et al. How people living with diabetes in Indonesia learn about their disease: a grounded theory study. PLoS One. 2019;14:e0212019. 30794570 10.1371/journal.pone.0212019 PMC6386238

[41] Rhee EJ. Diabetes in Asians. Endocrinol Metab. 2015;30:263–269. PubMed PMID: 26435131 10.3803/EnM.2015.30.3.263 PMC4595349 26435131

[42] Chan JC, Lau ES, Luk AO, et al. Premature mortality and comorbidities in young-onset diabetes: a 7-year prospective analysis. Am J Med. 2014;127:616–624. PubMed PMID: 24680795 Epub 2014 Apr 1. 24680795 10.1016/j.amjmed.2014.03.018

[43] Lascar N, Brown J, Pattison H, et al. Type 2 diabetes in adolescents and young adults. Lancet Diabetes Endocrinol. 2018;6:69–80. Epub 2017 Aug 30. PubMed PMID: 28847479. 28847479 10.1016/S2213-8587(17)30186-9

[44] Magliano DJ, Sacre JW, Harding JL, et al. Young-onset type 2 diabetes mellitus - implications for morbidity and mortality. Nat Rev Endocrinol. 2020;16:321–331. Epub 2020 Mar 24. PubMed PMID: 32203408. 32203408 10.1038/s41574-020-0334-z

[45] Visaria J, Iyer NN, Raval A, et al. Incidence and prevalence of microvascular and macrovascular diseases and all-cause mortality in type 2 diabetes mellitus: a 10-year study in a US commercially insured and medicare advantage population. Clin Ther. 2019;41:1522–36.e1. PubMed PMID: 31196656 Epub 2019 Jun 15. 31196656 10.1016/j.clinthera.2019.05.012

[46] Yeung RO, Zhang Y, Luk A, et al. Metabolic profiles and treatment gaps in young-onset type 2 diabetes in Asia (the JADE programme): a cross-sectional study of a prospective cohort. Lancet Diabetes Endocrinol. 2014;2:935–943.Epub 2014 Aug 2. PubMed PMID: 25081582. 2 25081582 10.1016/S2213-8587(14)70137-8

[47] Rao Kondapally Seshasai S, Kaptoge S, Thompson A, et al. Diabetes mellitus, fasting glucose, and risk of cause-specific death. N Engl J Med. 2011;364:829–841. PubMed PMID: 21366474; PubMed Central PMCID: PMCPMC4109980 Epub 2011 Mar 04. 21366474 10.1056/NEJMoa1008862 PMC4109980

[48] Hart JT. Rule of halves: implications of increasing diagnosis and reducing dropout for future workload and prescribing costs in primary care. Br J Gen Pract. 1992;42:116–119. PubMed PMID: 1493028 1493028 PMC1371996

[49] Jaacks LM, Vandevijvere S, Pan A, et al. The obesity transition: stages of the global epidemic. Lancet Diabetes Endocrinol. 2019;7:231–240. PubMed PMID: 30704950; PubMed Central PMCID: PMCPMC7360432 Epub 2019 Feb 2. 30704950 10.1016/S2213-8587(19)30026-9 PMC7360432

[50] Sacerdote C, Ricceri F, Rolandsson O, et al. Lower educational level is a predictor of incident type 2 diabetes in European countries: the EPIC-InterAct study. Int J Epidemiol. 2012;41:1162–1173. PubMed PMID: 22736421 Epub 2012 Jun 28. 22736421 10.1093/ije/dys091

[51] Seiglie JA, Marcus M-E, Ebert C, et al. Diabetes prevalence and its relationship with education, wealth, and BMI in 29 low- and middle-income countries. Diabetes Care. 2020;43:767–775. 32051243 10.2337/dc19-1782 PMC7085810

[52] Flies EJ, Mavoa S, Zosky GR, et al. Urban-associated diseases: candidate diseases, environmental risk factors, and a path forward. Environ Int. 2019;133:105187. 31648161 10.1016/j.envint.2019.105187

[53] Barker D, Eriksson J, Forsén T, et al. Fetal origins of adult disease: strength of effects and biological basis. Int J Epidemiol. 2002;31:1235–1239. 12540728 10.1093/ije/31.6.1235

[54] Hales CN, Barker DJP. The thrifty phenotype hypothesis: type 2 diabetes. Br Med Bull. 2001;60:5–20. 11809615 10.1093/bmb/60.1.5

[55] Yajnik CS. Early life origins of insulin resistance and type 2 diabetes in India and other Asian countries. J Nutr. 2004;134:205–210. Epub 2004 Jan 6. PubMed PMID: 14704320. 14704320 10.1093/jn/134.1.205

[56] Tamayo T, Christian H, Rathmann W. Impact of early psychosocial factors (childhood socioeconomic factors and adversities) on future risk of type 2 diabetes, metabolic disturbances and obesity: a systematic review. BMC Public Health. 2010;10:525. Epub 2010 Sept 3. PubMed PMID: 20809937; PubMed Central PMCID: PMCPMC2940917. 20809937 10.1186/1471-2458-10-525 PMC2940917

[57] Demakakos P, Marmot M, Steptoe A. Socioeconomic position and the incidence of type 2 diabetes: the ELSA study. Eur J Epidemiol. 2012;27:367–378. 22539241 10.1007/s10654-012-9688-4

[58] Tsenkova V, Pudrovska T, Karlamangla A. Childhood socioeconomic disadvantage and prediabetes and diabetes in later life: a study of biopsychosocial pathways. Psychosom Med. 2014;76:622–628. PubMed PMID: 25272201 25272201 10.1097/PSY.0000000000000106 PMC4229367

[59] Sutanegara D, Budhiarta AA. The epidemiology and management of diabetes mellitus in Indonesia. Diabetes Res Clin Pract. 2000;50:S9–s16. Epub 2000 Oct 12. PubMed PMID: 11024578. 11024578 10.1016/s0168-8227(00)00173-x

[60] Directorate General of Non-Communicable Disease Prevention and Control. Program P2PTM dan indikator [non-communicable disease prevention and control program and indicators]. Jakarta Indonesia: Ministry of Health Indonesia; 2016 cited 2021 Apr 17. Available from: http://p2ptm.kemkes.go.id/profil-p2ptm/latar-belakang/program-p2ptm-dan-indikator.

[61] Pranandari LL, Arso SP, and Fatmasari EY. [Noncommunicable disease management post program implementation analysis at sub-district Banguntapan in Bantul district]. Journal Kesehatan Masyarakat. 2017;5:4.

[62] Primiyani Y, Masrul M, and Hardisman H. [Noncommunicable disease management post program implementation analysis in Solok City]. Journal Kesehatan Andalas. 2019;8:2.

[63] Baird J, Jacob C, Barker M, et al. Developmental origins of health and disease: a lifecourse approach to the prevention of non-communicable diseases. Healthcare. 2017;5:14. PubMed PMID: 28282852; PubMed Central PMCID: PMCPMC5371920 Epub 2017 Mar 12. 10.3390/healthcare5010014 PMC5371920 28282852

[64] Leighton E, Sainsbury CA, Jones GC. A practical review of C-Peptide testing in diabetes. Diabetes Ther. 2017;8:475–487.Epub 2017 May 8. PubMed PMID: 28484968. 8 28484968 10.1007/s13300-017-0265-4 PMC5446389

[65] Dejkhamron P, Santiprabhob J, Likitmaskul S, et al. Type 1 diabetes management and outcomes: a multicenter study in Thailand. J Diabetes Investig. 2021;12:516–526. 10.1111/jdi.13390 PMC8015826 32815278

[66] Patterson CC, Karuranga S, Salpea P, et al. Worldwide estimates of incidence, prevalence and mortality of type 1 diabetes in children and adolescents: results from the international diabetes federation diabetes atlas, 9th edition. Diabetes Res Clin Pract. 2019; 157. Epub 2019 Sep 10. 10.1016/j.diabres.2019.107842. PubMed PMID: 107842. 31518658

[67] Pulungan AB, Fadiana G, Annisa D. Type 1 diabetes mellitus in children: experience in Indonesia. Clin Pediatr Endocrinol. 2021;30:11–18. Epub 2021 Jan 5. PubMed PMID: 33446947. 33446947 10.1297/cpe.30.11 PMC7783121

[68] World Health Organization. Use of glycated haemoglobin (HbA1c) in the diagnosis of diabetes mellitus: abbreviated report of a WHO consultation. Geneva: World Health Organization; 2011. cited 2021 Jan 18. Available from: https://www.ncbi.nlm.nih.gov/books/NBK304267/pdf/Bookshelf_NBK304267.pdf. 26158184

