# Supplementary material for: Diabetes prevalence and risk factors of early-onset adult diabetes: results from the Indonesian family life survey
Source: Glob Health Action. 2021 Dec 13;14(1):2001144. doi: 10.1080/16549716.2021.2001144 (PMC8676618; doi:10.1080/16549716.2021.2001144)
Supplement: Supplemental Material [file ZGHA_A_2001144_SM1918.docx]

**Supplementary materials**

Table S1. HbA1c test results on selected participants in ILFS 5 independent of self-reported diabetes.

|  | **HbA1c test results** | | | | | | | | | | | | | | | | |  | | | |
| --- | --- | --- | --- | --- | --- | --- | --- | --- | --- | --- | --- | --- | --- | --- | --- | --- | --- | --- | --- | --- | --- |
| **Age at IFLS 5** | **< 5.7%** | | | | **5.7 - < 6.5%** | | | | **6.5 - < 7%** | | | | **≥ 7%** | | | | | **Total** | | | |
| **15-19yo** | 436 | ( | 82.9% | ) | 80 | ( | 15.2% | ) | 8 | ( | 1.5% | ) | 2 | ( | 0.4% | ) | 526 | | ( | 100.0% | ) |
| **20-30yo** | 1104 | ( | 80.5% | ) | 237 | ( | 17.3% | ) | 10 | ( | 0.7% | ) | 20 | ( | 1.5% | ) | 1371 | | ( | 100.0% | ) |
| **31-40yo** | 963 | ( | 70.0% | ) | 341 | ( | 24.8% | ) | 35 | ( | 2.5% | ) | 36 | ( | 2.6% | ) | 1375 | | ( | 100.0% | ) |
| **41-50yo** | 518 | ( | 59.8% | ) | 262 | ( | 30.3% | ) | 25 | ( | 2.9% | ) | 61 | ( | 7.0% | ) | 866 | | ( | 100.0% | ) |
| **51-60yo** | 488 | ( | 51.9% | ) | 310 | ( | 32.9% | ) | 44 | ( | 4.7% | ) | 99 | ( | 10.5% | ) | 941 | | ( | 100.0% | ) |
| **> 60yo** | 880 | ( | 54.3% | ) | 520 | ( | 32.1% | ) | 77 | ( | 4.8% | ) | 143 | ( | 8.8% | ) | 1620 | | ( | 100.0% | ) |
| **Total** | 4389 | ( | 65.5% | ) | 1750 | ( | 26.1% | ) | 199 | ( | 3.0% | ) | 361 | ( | 5.4% | ) | 6699 | | ( | 100.0% | ) |

Table S2. HbA1c test results among self-reported diabetic participants grouped by self-reported prescribed medicine consumption and age at IFLS 5.

|  | |  | HbA1c test results | | | | |
| --- | --- | --- | --- | --- | --- | --- | --- |
|  | |  | **< 5.7%** | **5.7 - < 6.5%** | **6.5 - < 7%** | **≥ 7%** | **NA** |
| Taking prescribed medicine | Yes | **15-19yo** | 0 | 1 | 0 | 0 | 1 |
|  |  | **20-30yo** | 1 | 0 | 1 | 0 | 17 |
|  |  | **31-40yo** | 4 | 2 | 0 | 0 | 54 |
|  |  | **41-50yo** | 3 | 1 | 0 | 6 | 68 |
|  |  | **51-60yo** | 5 | 2 | 1 | 14 | 86 |
|  |  | **> 60yo** | 4 | 12 | 4 | 16 | 77 |
|  | No | **15-19yo** | 0 | 0 | 0 | 0 | 0 |
|  |  | **20-30yo** | 0 | 0 | 0 | 0 | 4 |
|  |  | **31-40yo** | 1 | 0 | 1 | 1 | 29 |
|  |  | **41-50yo** | 0 | 0 | 0 | 7 | 72 |
|  |  | **51-60yo** | 2 | 5 | 1 | 30 | 115 |
|  |  | **> 60yo** | 2 | 6 | 2 | 36 | 102 |

Table S3. Results of univariable Cox regression sensitivity analyses with different sub-cohorts.

| **Outcome:**  **Early-onset diabetes** | **Levels** | **HR (Univariable)** | | |
| --- | --- | --- | --- | --- |
|  |  | **Sub-cohort**  **(Table 2)**  **N = 31 610** | **Only tested individuals**  **N = 6 172** | **Only self-reported cases^*^**  **N = 31 610** |
| **Sex** | Female | 1 | 1 | 1 |
|  | Male | 1.03 (0.81-1.30, p=0.828) | 1.22 (0.85-1.75, p=0.280) | 0.97 (0.72-1.31, p=0.848) |
| **Education level** | Low | 1 | 1 | 1 |
|  | Middle | 2.67 (2.01-3.55, **p<0.001**) | 4.01 (2.59-6.20, **p<0.001**) | 2.51 (1.77-3.58, **p<0.001**) |
|  | High | 3.49 (2.45-4.98, **p<0.001**) | 4.46 (2.54-7.83, **p<0.001**) | 3.77 (2.45-5.78, **p<0.001**) |
| **General health in childhood** | Poor | 1.00 (0.63-1.58, p=0.999) | 0.94 (0.48-1.84, p=0.849) | 0.94 (0.52-1.70, p=0.829) |
|  | Fair | 0.83 (0.61-1.13, p=0.237) | 0.80 (0.51-1.25, p=0.326) | 0.82 (0.56-1.22, p=0.332) |
|  | Good | 1 | 1 | 1 |
|  | Very good | 0.95 (0.66-1.35, p=0.762) | 0.73 (0.41-1.30, p=0.283) | 0.98 (0.63-1.52, p=0.931) |
|  | Excellent | 1.34 (0.83-2.16, p=0.232) | 1.16 (0.55-2.44, p=0.699) | 1.48 (0.83-2.63, p=0.180) |
| **Ever experienced starvation in childhood** | No | 1 | 1 | 1 |
|  | Yes | 0.67 (0.42-1.05, p=0.083) | 0.39 (0.17-0.89, p=0.026) | 0.85 (0.51-1.43, p=0.547) |
| **Childhood socio-economic level** | Low | 0.72 (0.49-1.05, p=0.088) | 0.79 (0.45-1.37, p=0.397) | 0.64 (0.39-1.05, p=0.077) |
|  | Middle | 1 | 1 | 1 |
|  | High | 0.65 (0.49-0.85, **p=0.002**) | 0.68 (0.45-1.02, p=0.063) | 0.59 (0.41-0.83, **p=0.002**) |
| **Residence area in childhood** | Rural | 1 | 1 | 1 |
|  | Urban | 1.67 (1.24-2.25, **p=0.001**) | 1.09 (0.70-1.71, p=0.690) | 2.64 (1.77-3.94, **p<0.001**) |
|  | Unknown | 1.59 (1.17-2.15, **p=0.003**) | 1.85 (1.20-2.86, **p=0.006**) | 2.35 (1.56-3.54, **p<0.001**) |

^*^ *Diabetes patients detected by HbA1c measurement was treated as non-diabetic.*

*Table S4. Multivariable Cox regression results comparison between the total sub-cohort (main analysis) and sub-cohorts excluding individuals who (a) had BMI ≤ 23 kg/m^2^ and were taking insulin or (b) had BMI ≤ 23 kg/m^2^.*

| **Outcome:**  **Early-onset diabetes** | **Levels** | **HR** *^§^***(Multivariable)^°^** | | |
| --- | --- | --- | --- | --- |
|  |  | **Total sub-cohort**  **(257 cases)** | **Participants with BMI**  **≤ 23kg/m^2^ and insulin medication excluded**  **(252 cases)^a^** | **Participants with BMI**  **≤ 23kg/m^2^ excluded**  **(179 cases)^b^** |
| **Sex** | Female | 1 | 1 | 1 |
|  | Male | 0.98 (0.77-1.25, p=0.883) | 0.99 (0.77-1.26, p=0.907) | 0.75 (0.56-1.00, p=0.054) |
| **Education level** | Low | 1 | 1 | 1 |
|  | Middle | 1.34 (0.95-1.87, p=0.094) | 1.39 (0.99-1.96, p=0.061) | 1.46 (0.98-2.17, p=0.061) |
|  | High | 1.67 (1.10-2.52, **p=0.015**) | 1.76 (1.16-2.67, **p=0.008**) | 2.07 (1.30-3.31, **p=0.002**) |
| **General health in childhood** | Poor | 1.11 (0.69-1.80, p=0.666) | 1.15 (0.71-1.87, p=0.576) | 0.94 (0.49-1.79, p=0.849) |
|  | Fair | 0.82 (0.60-1.11, p=0.199) | 0.84 (0.62-1.15, p=0.281) | 0.81 (0.56-1.18, p=0.273) |
|  | Good | 1 | 1 | 1 |
|  | Very good | 0.85 (0.60-1.21, p=0.375) | 0.88 (0.62-1.25, p=0.463) | 0.88 (0.58-1.33, p=0.554) |
|  | Excellent | 1.48 (0.92-2.39, p=0.105) | 1.37 (0.83-2.26, p=0.213) | 1.59 (0.92-2.75, p=0.099) |
| **Ever experienced starvation in childhood** | No | 1 | 1 | 1 |
|  | Yes | 1.23 (0.76-1.99, p=0.397) | 1.27 (0.78-2.06, p=0.333) | 1.52 (0.87-2.64, p=0.137) |
| **Childhood socio-economic level** | Low | 0.87 (0.58-1.29, p=0.488) | 0.82 (0.54-1.24, p=0.353) | 0.94 (0.59-1.50, p=0.802) |
|  | Middle | 1 | 1 | 1 |
|  | High | 0.82 (0.62-1.09, p=0.165) | 0.81 (0.61-1.07, p=0.132) | 0.76 (0.53-1.07, p=0.114) |
| **Residence area in childhood** | Rural | 1 | 1 | 1 |
|  | Urban | 1.51 (1.08-2.10, **p=0.016**) | 1.48 (1.06-2.06, **p=0.023**) | 1.70 (1.12-2.59, **p=0.012**) |
|  | Unknown | 0.97 (0.71-1.33, p=0.853) | 0.96 (0.70-1.31, p=0.780) | 1.12 (0.76-1.66, p=0.570) |

Table S5. Indonesian Family Life Survey: *Multivariable Cox regression analyses with (#) and without earlier BMI (main analysis), Indonesia <1993 - 2014>.*

| **Outcome:**  **Early-onset Diabetes** | **Levels** | **n (%)** | **HR**  **(Multivariable)** | **HR**  **(Multivariable + earlier BMI)^#^** |
| --- | --- | --- | --- | --- |
| **Sex** | Female | 16534 (52.3) | 1 | 1 |
|  | Male | 15076 (47.7) | 0.98 (0.77-1.25, p=0.883) | 1.16 (0.91-1.49, p=0.240) |
| **Education level** | Low | 12180 (38.6) | 1 | 1 |
|  | Middle | 14923 (47.3) | 1.34 (0.95-1.87, p=0.094) | 1.28 (0.91-1.80, p=0.151) |
|  | High | 4462 (14.1) | 1.67 (1.10-2.52, **p=0.015**) | 1.52 (1.01-2.29, **p=0.047**) |
| **General health in childhood** | Poor | 1465 (5.2) | 1.11 (0.69-1.80, p=0.666) | 1.19 (0.73-1.93, p=0.488) |
|  | Fair | 5082 (17.9) | 0.82 (0.60-1.11, p=0.199) | 0.85 (0.62-1.15, p=0.286) |
|  | Good | 11319 (39.9) | 1 | 1 |
|  | Very good | 8294 (29.3) | 0.85 (0.60-1.21, p=0.375) | 0.86 (0.60-1.22, p=0.386) |
|  | Excellent | 2187 (7.7) | 1.48 (0.92-2.39, p=0.105) | 1.46 (0.91-2.36, p=0.119) |
| **Ever experienced starvation in childhood** | No | 25924 (91.5) | 1 | 1 |
|  | Yes | 2423 (8.5) | 1.23 (0.76-1.99, p=0.397) | 1.20 (0.75-1.94, p=0.443) |
| **Childhood socio-economic level** | Low | 4041 (14.3) | 0.87 (0.58-1.29, p=0.488) | 0.90 (0.61-1.35, p=0.617) |
|  | Middle | 13736 (48.5) | 1 | 1 |
|  | High | 10571 (37.3) | 0.82 (0.62-1.09, p=0.165) | 0.85 (0.64-1.12, p=0.254) |
| **Residence area in childhood** | Rural | 11734 (37.1) | 1 | 1 |
|  | Urban | 9223 (29.2) | 1.51 (1.08-2.10, **p=0.016**) | 1.39 (1.00-1.94, **p=0.050**) |
|  | Unknown | 10653 (33.7) | 0.97 (0.71-1.33, p=0.853) | 0.89 (0.65-1.23, p=0.486) |
| **BMI (earliest)** | < 18.5kg/m2 | 3935 (12.4) | NA | 1.24 (0.77-2.01, p=0.376) |
|  | 18.5 – < 23kg/m2 | 14528 (46.0) |  | 1 |
|  | 23 – < 27.5kg/m2 | 8285 (26.2) |  | 2.17 (1.59-2.94, **p<0.001**) |
|  | ≥ 27.5kg/m2 | 3003 (9.5) |  | 3.30 (2.28-4.77, **p<0.001**) |
|  | Unknown | 1859 (5.9) |  | 3.78 (2.22-6.43, **p<0.001**) |
